# Subverting colonization resistance: *Salmonella* pathogenesis at the interface of microbiota, metabolism and inflammasome

**DOI:** 10.1080/21505594.2026.2745323

**Published:** 2026-10-16

**Authors:** Zhixin Gao, Zheng Li, Jianyang Cui, Qing Zhang, Yanyan Jia, Ke Ding, Ke Shang, Songbiao Chen

**Affiliations:** a Laboratory of Functional Microbiology and Animal Health, College of Animal Science and Technology, Henan University of Science and Technology, Luoyang, P.R. China; b Luoyang Key Laboratory of Live Carrier Biomaterial and Animal Disease Prevention and Control, Henan University of Science and Technology, Luoyang, P.R. China; c The Key Lab of Animal Disease and Public Health, Henan University of Science and Technology, Luoyang, P.R. China; d Ministry of Education Key Laboratory for Animal Pathogens and Biosafety, Zhengzhou, P.R.China

**Keywords:** *Salmonella*, microbiota dysbiosis, inflammasome activation, colonization resistance

## Abstract

The intestinal tract is a key immune organ and microecological system, where colonization resistance (CR) mediated by gut microbiota prevents pathogen invasion. *Salmonella* disrupts CR via type VI and type III secretion systems (T6SS/T3SS), inducing dysbiosis, inflammasome-driven inflammation, and intestinal barrier damage. This creates a self-reinforcing cycle:dysbiosis alters microbial metabolites, sustains inflammation, and enhances pathogen fitness, favoring chronic infection. This review integrates molecular mechanisms of microbiota disruption, inflammasome activation, and metabolic imbalance to explain how *Salmonella* overcomes CR. We also highlight microbiota-based countermeasures, including probiotics, natural antimicrobials, and phage therapy, as promising strategies to restore gut homeostasis.

## Introduction

As the body’s largest immune organ and the primary site of microecological colonization, the maintenance of intestinal homeostasis heavily relies on the dynamic balance between the host’s innate immune defense system and the gut microbiota [1,2]. Once this balance is disrupted, gut dysbiosis can directly induce or exacerbate the onset and progression of various gastrointestinal diseases [3,4].

Intestinal “colonization resistance” (CR) refers to the protective barrier formed by the gut commensal microbiota, which resists the invasion and colonization of exogenous pathogenic bacteria, serving as the body’s first line of defense and playing a key role in anti-infective immunity [5,6]. A critical step for enteric pathogenic microorganisms to achieve systemic infection lies in their ability to breach the intestinal physical mucosal barrier and subsequently occupy the dominant ecological niche within the gut [7–9]. Our previous studies have found that after invading the host via the oral-intestinal route, *Salmonella enterica* serovar Typhimurium (*S*. Typhimurium) utilizes its T6SS to enhance bacterial tolerance to acidic, alkaline, and saline environments [10], alters gut microbiota richness, induces the expression of pro-inflammatory factors such as GBP2b, IFNB1, IL-6, NLRP3, NOS2, and TNF-α, and disrupts the intestinal physical barrier by affecting the expression of mucins including ZO-1 and occludin, thereby occupying a favorable ecological niche [11]. Further studies have also found that *Salmonella* can activate inflammatory pathways by inducing dysbiosis and alter metabolites to promote inflammatory bowel disease (IBD), thereby establishing a vicious cycle of “infection colonization – microbiota disturbance – inflammation exacerbation” [12–14](Figure 1). These studies have confirmed that after entering intestinal epithelial cells, *Salmonella* engages in complex interactions with the gut microbiota, reshaping the abundance of the normal microbiota and modulating the production of metabolites to regulate bacterial and host functions.

**Figure 1.**
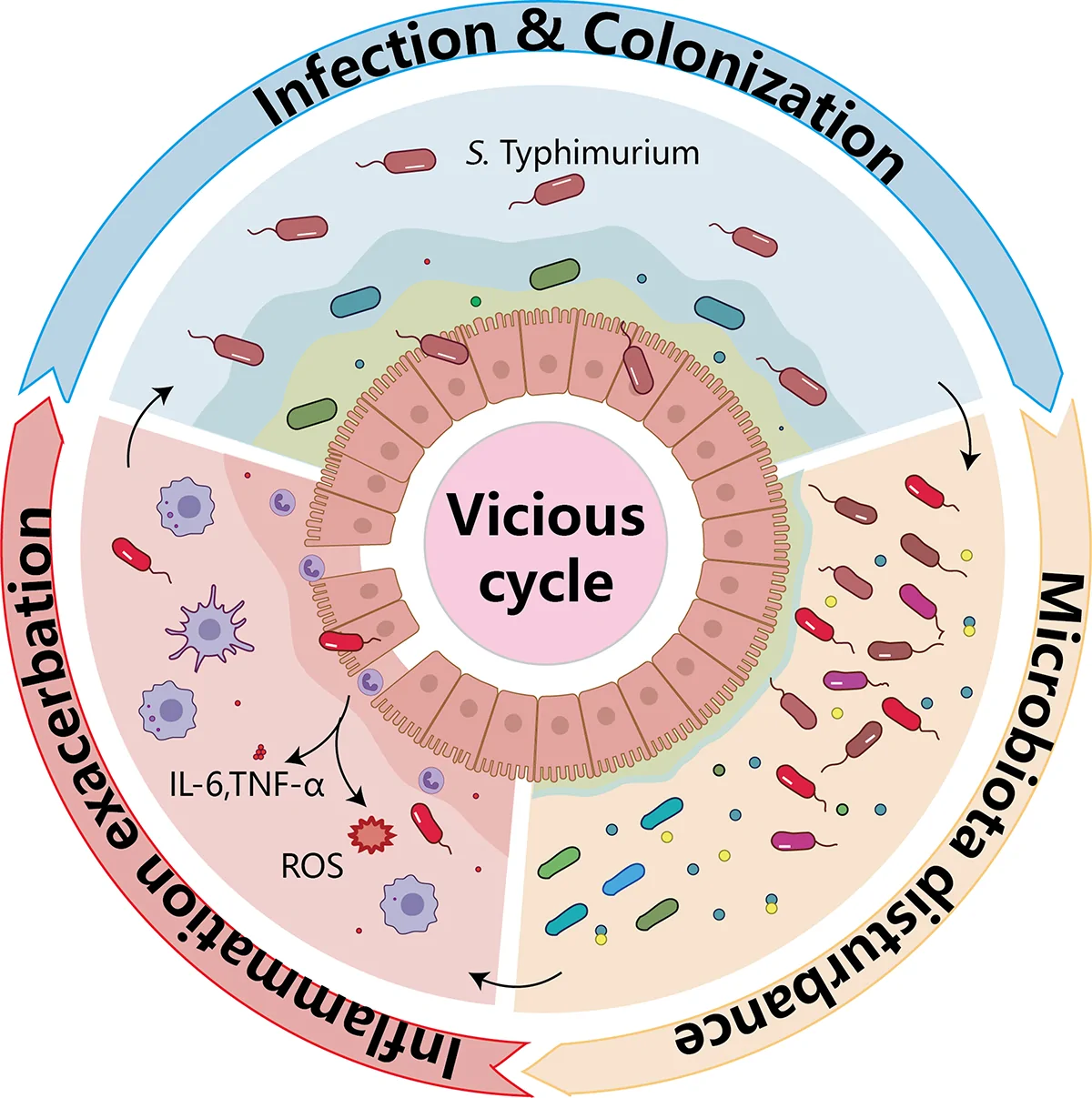
A vicious cycle of “infection colonization – microbiota disturbance – inflammation exacerbation”. *S*. Typhimurium disrupts epithelial barrier integrity to establish colonization, inducing gut microbiota dysbiosis and loss of colonization resistance. Subsequent inflammatory responses promote immune activation, cytokine IL-6, TNF-α and ROS production, leading to epithelial damage and creating a favorable niche for *Salmonella* expansion, thereby forming a self-reinforcing vicious cycle.

This review aims to elucidate how *Salmonella* disrupts intestinal microecological balance and modulates the production and alterations of gut microbial metabolites to overcome intestinal CR, with the goal of providing theoretical foundations and research perspectives for identifying novel intervention targets for the prevention and control of *Salmonella* infection. This review provides a comprehensive framework for understanding how *Salmonella* overcomes intestinal CR and highlights potential microbiota-based interventions, including probiotics, natural antimicrobial agents, and bacteriophage therapies.

## The gut microbiota acts as a crucial barrier for the maintenance of host health

The gut microbiota refers to the complex microbial community residing in the digestive tract of animals. Compared to other parts of the body, the gut microbiota harbors the largest microbial population and the greatest species diversity, including bacteria, viruses, and some eukaryotes [15]. This community rapidly establishes itself in the digestive tract after birth and consists of more than 50 different phyla, encompassing over 1,500 species [16]. Among these, *Bacteroides* can serve as a predominant core genus in certain human populations and animal models; however, its abundance varies depending on host species, geographic location, dietary factors, and other environmental influences. Other major groups include *Proteobacteria*, *Firmicutes*, *Actinobacteria*, and *Blautia* [17].

The functions of the gut microbiota span several essential biological processes, including nutrient metabolism, barrier defense, immune regulation, and neural communication, playing an irreplaceable role in maintaining host homeostasis. In nutrient metabolism, gut microbes secrete various digestive enzymes that facilitate the fermentation of carbohydrates and complex polysaccharides, which the host itself cannot digest, generating short-chain fatty acids (SCFAs) and other small metabolites that provide a vital energy source for intestinal epithelial cells (Figure 2(A)) [18,19]. Moreover, these microbes synthesize B vitamins, vitamin K, and amino acids, compensating for the host’s inability to produce these essential nutrients independently [20]. In terms of barrier defense, beneficial bacteria compete for nutrients and adhesion sites, inhibiting the colonization and excessive proliferation of pathogenic bacteria, thereby maintaining the structural balance of the gut microbiota (Figure 2(B)). Their metabolic products also promote the formation of tight junctions (TJs) between intestinal epithelial cells, enhancing the integrity of the gut physical barrier, reducing the entry of pathogens, toxins, and undigested macromolecules into the bloodstream, and thereby lowering the risk of inflammation caused by increased intestinal permeability [21,22]. In immune regulation, the gut microbiota serves as the largest immune stimulus source for the body. By interacting with the intestinal mucosal immune system, it promotes the development and maturation of immune cells, regulates cytokine secretion, and helps the host distinguish between beneficial microbes and harmful pathogens. This delicate balance between immune tolerance and immune response prevents excessive inflammation or immune dysregulation (Figure 2(C)) [23,24]. Regarding neural communication, the gut microbiota communicates bidirectionally with the central nervous system (CNS) through the gut–brain axis (GBA) (Figure 2(D)). Microbial metabolites, including SCFAs and neuroactive molecules, transmit signals to the brain via the bloodstream, vagus nerve, and immune pathways, influencing appetite, mood, and cognition. Meanwhile, the brain regulates intestinal function through neural, endocrine, and immune pathways, with the Hypothalamic–Pituitary–Adrenal Axis (HPA axis) and autonomic nervous system (ANS) modulating gut motility, barrier integrity, immunity, and microbial composition. This bidirectional interaction contributes to maintaining host metabolic, immune, and neurological homeostasis [25–27].

**Figure 2.**
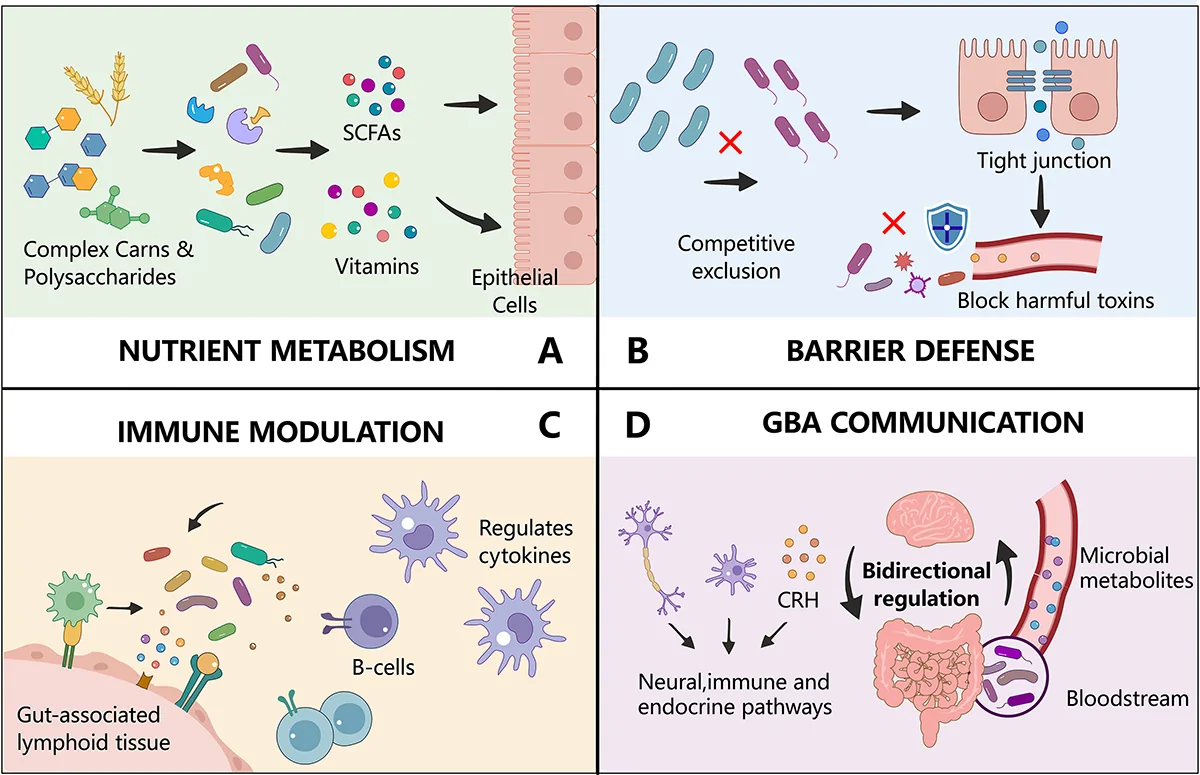
Overview of gut microbiota composition and functions. (A) Nutrient metabolism-enzymatic fermentation of complex carbohydrates and polysaccharides to produce vitamins for epithelial cells; (B) Barrier defense–competitive exclusion of pathogens and maintenance of the mucus layer; (C) Immune modulation-regulation of cytokine production and immune tolerance; (D) Bidirectional GBA communication: gut microbiota-derived metabolites regulate brain activity via the bloodstream, whereas the brain controls intestinal function through neural, endocrine, and immune pathways.

## *Salmonella* induces intestinal dysbiosis

In recent years, the T6SS, recognized as one of the key virulence determinants of *Salmonella*, has attracted increasing attention due to its role in reshaping the host microbiota to establish a competitive advantage during infection [28]. Emerging evidence indicates that the T6SS functions as a sophisticated “molecular weapon” that drives microbiota dysbiosis through targeted elimination of competing microbes and precise ecological regulation [29]. Structurally resembling an inverted bacteriophage tail, the T6SS employs a contractile sheath to propel a spike complex composed of proteins such as Hcp and VgrG into target cells, thereby delivering diverse effector molecules. These effectors include peptidoglycan hydrolases (e.g. Tae4) that degrade bacterial cell walls, phospholipases that disrupt membrane integrity, and nucleases capable of degrading DNA or RNA [30,31]. The functional precision of the *Salmonella* T6SS is further reflected by the specialization of its core components. *S*. Typhimurium encodes three distinct Hcp proteins (Hcp1, Hcp2, and Hcp3), which exhibit non-redundant functions. Recent studies have demonstrated that deletion of different *hcp* genes results in distinct alterations in the intestinal microbial community. For instance, infection with the wild-type strain induces a pronounced expansion of the *Escherichia-Shigella* genus, a hallmark of inflammation-associated dysbiosis, whereas mutants lacking specific *Hcp* components cause comparatively limited microbiota perturbations [32]. Moreover, intestinal environmental cues, including bile salts, pH fluctuations, and elevated magnesium concentrations, serve as critical signals for T6SS activation, ensuring that this system is deployed only under appropriate conditions [33].

In addition to T6SS-mediated microbial competition, *S*. Typhimurium can suppress competing commensal bacteria through the T3SS encoded by *Salmonella* pathogenicity island 1 (SPI-1). In both cellular and murine models, through this secretion apparatus, *Salmonella* delivers effector proteins such as SopB and SopE into host cells, promoting membrane ruffling and facilitating the formation of Salmonella-containing vacuoles (SCVs) [34]. Meanwhile, in a comparative infection experiment in which C57BL/6 mice were orally inoculated with wild-type *Salmonella* and chloramphenicol-resistant *ΔgogB Salmonella* strains, the secreted effector protein GogB was shown to suppress intracellular inflammatory signaling pathways and reduce macrophage bactericidal activity, thereby promoting the long-term survival and stable colonization of *Salmonella* within the host [35,36].

Meanwhile, *Salmonella* engages in intense competition with symbionts for essential trace elements by utilizing its high-affinity metal transport systems. In inflammatory environments, the host secretes lipid transport protein-2 to chelate iron carriers of common bacteria, whereas *S*. Typhimurium synthesizes salmochelin via the *iroB* gene-encoded glycosyltransferase, escaping recognition by lipid transport protein-2 and securing preferential access to iron [37,38]. In a mouse in vivo experimental model, *S*. Typhimurium also secretes antimicrobial effector proteins (e.g. RHs, Ssp) through the T6SS, inducing depolarization of symbiont cell membranes and causing the release of intracellular zinc ions. Subsequently, *Salmonella* efficiently sequesters these zinc ions using its own ZnuABC zinc uptake system, thereby inhibiting the growth of symbionts [39]. This nutrient-competition-based mechanism allows *Salmonella* to establish a significant competitive advantage in the resource-limited gut environment.

The disruption of the intestinal barrier further exacerbates dysbiosis. Specifically, *Salmonella* effector proteins SopB and SopE activate host Rho family GTPases (e.g. Cdc42 and Rac1), triggering actin cytoskeleton rearrangement and interfering with the normal localization of TJ proteins Occludin and Claudin-1 [40,41]. Damage to the TJs increases intestinal permeability, allowing trace amounts of oxygen to penetrate the previously anaerobic gut lumen, which suppresses the growth of strict anaerobes like *Clostridium*, important for maintaining intestinal homeostasis. For example, in a *S*. Typhimurium infection model using CBA/J mice, epithelial hypoxia in the control group maintained the oxygen concentration in the cecal lumen at approximately 0.6% O_2_, creating a near-anaerobic environment. In contrast, infection-induced loss of epithelial hypoxia resulted in a marked increase in luminal oxygen availability, with oxygen levels in the cecal lumen rising to approximately 4% O_2_, comparable to those measured in the cecal mucosa. Meanwhile, plasma proteins and cell debris also leak into the gut lumen, providing *Salmonella* with a rich nutritional source [42–44].

The strategic exploitation of the inflammatory response is central to *Salmonella*‘s reshaping of the gut microbiota. The strong inflammatory response triggered by *Salmonella* invasion of intestinal epithelium appears to be an expression of the host’s defense mechanisms but is cleverly co-opted by the pathogen to gain metabolic advantages. For example, in a murine colitis model, *S*. Typhimurium utilizes nitrate reductases NapABC, encoded by the *nap* gene cluster, to perform nitrate respiration in the inflammatory environment, thus obtaining additional energy for selective proliferation [45]. Alongside this process, ethanolamine released by sloughed intestinal epithelial cells is converted into carbon and nitrogen sources by *S*. Typhimurium through the ethanolamine utilization enzymes encoded by the *eut* gene cluster, providing crucial metabolic fuel in the inflammatory milieu [46]. These mechanisms work synergistically, allowing *Salmonella* to convert the host’s inflammatory response into a favorable metabolic advantage for its own growth, while symbionts unable to utilize these inflammatory by-products are gradually eliminated. The combined effect of these mechanisms ultimately leads to a decrease in microbiota diversity and structural alterations, resulting in dysbiosis [47,48].

## Molecular mechanisms of *Salmonella*-induced dysbiosis in disrupting intestinal CR via altered microbial metabolites

CR in the intestine represents a multidimensional defense system co-established by the host and its commensal microbiota. Its foundation extends beyond physical barriers and immune surveillance to encompass a chemically mediated ecological equilibrium governed by microbial metabolic networks. Key metabolites – including SCFAs, amino acid derivatives, bile acids products – collectively form a chemical barrier that suppresses pathogen expansion, nourishes epithelial cells, and modulates immune responses [49]. *S*. Typhimurium, a prototypical invasive enteric pathogen, actively disrupts this balance by inducing widespread dysbiosis and systematically reshaping the intestinal metabolic landscape. In doing so, it converts a chemical environment that is normally unfavorable to pathogens into a niche that supports its survival, proliferation, and virulence, ultimately overcoming intestinal CR [50].

### Alterations in short-chain fatty acids exacerbate intestinal inflammation

SCFAs, particularly acetate, propionate, and butyrate, represent the central effectors of intestinal CR [51]. *Salmonella* infection, through both direct and indirect mechanisms, markedly reduces SCFA production and disrupts their functions, thereby dismantling this critical chemical barrier (Figure 3(A)). Acetate is the most abundant SCFA in the gut. Its primary role is to lower luminal pH, creating an acidic environment that is unfavorable for the growth of many pathogens while imposing stress on bacterial intracellular pH homeostasis [52]. During pathogen colonization, inflammation-induced fluid secretion dilutes luminal contents, significantly decreasing the effective local concentrations of acetate and other SCFAs. Concurrently, infection-associated dysbiosis destabilizes acetate-producing communities, such as *Bacteroides* spp., further reducing acetate availability. This weakens the nonspecific antimicrobial barrier and provides a window for early *S*. Typhimurium survival and expansion [53].

**Figure 3.**
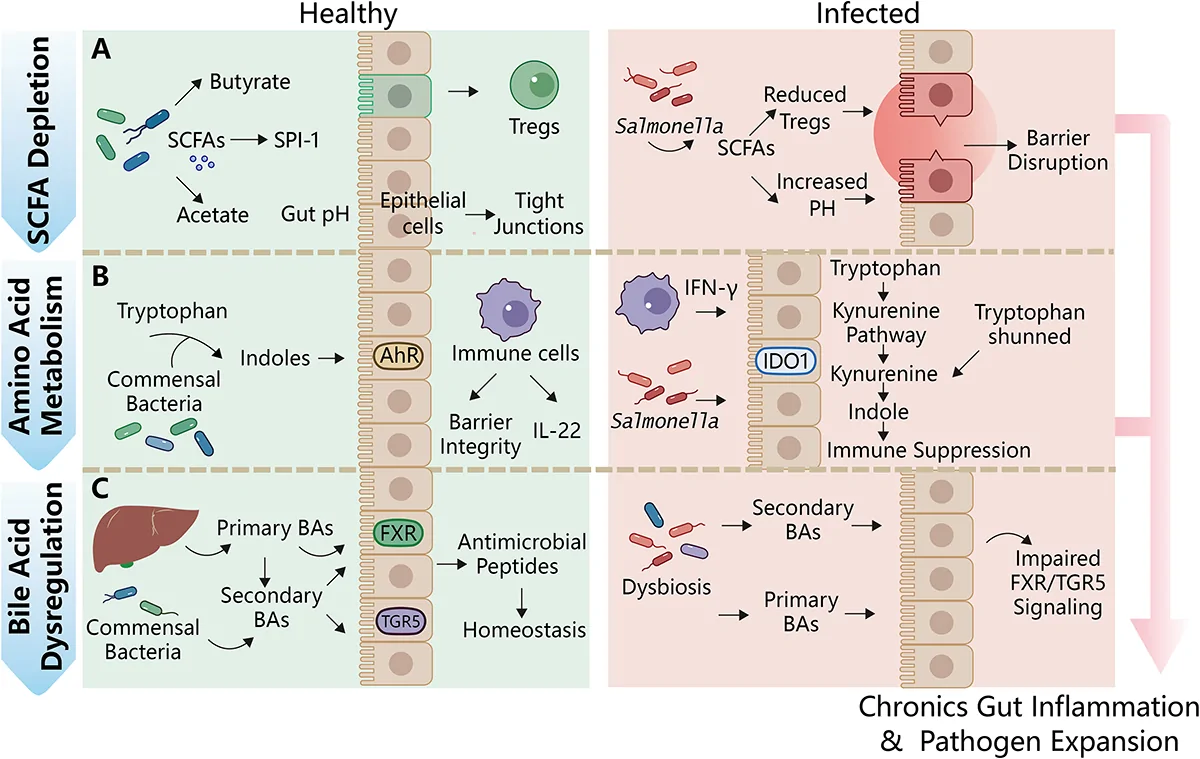
Schematic diagram of *Salmonella* -induced metabolic dysregulation and gut inflammation. (A) SCFA depletion and barrier loss. Commensal bacteria produce SCFAs that maintain TJs, support Tregs, and suppress *Salmonella* virulence via SPI-1 inhibition. *Salmonella* infection increases luminal pH, reducing SCFAs, enhancing virulence, and disrupting barrier integrity. (B) Amino acid metabolism shift (tryptophan focus). Under homeostasis, commensal bacteria convert tryptophan into indoles, activating AhR to induce IL-22 and promote mucosal defense. During infection, IFN-γ upregulates IDO1, shifting metabolism toward the kynurenine pathway, leading to immune suppression and reduced indole production. (C) Bile acid dysregulation and signaling disruption. Secondary bile acids activate FXR/TGR5 signaling to support antimicrobial peptide production and immune homeostasis. *Salmonella* infection causes accumulation of conjugated primary bile acids, impairing FXR/TGR5 signaling, reducing antimicrobial defense, and exacerbating inflammation. These metabolic disruptions collectively drive chronic gut inflammation and pathogen expansion.

Propionate not only exerts direct antimicrobial effects but also possesses two key anti-*Salmonella* functions. First, it acts as a potent inhibitor of virulence gene expression. Upon entering *Salmonella* cells, propionate suppresses the translation of the master virulence regulator HilD, thereby downregulating genes within *Salmonella* SPI-1 and significantly impairing epithelial invasion [54]. Second, propionate exerts systemic anti-inflammatory effects by signaling through G protein–coupled receptors, such as GPR43, to restrain excessive activation of neutrophils and macrophages. However, pathogen invasion depletes propionate-producing bacteria, leading to reduced propionate levels. Metabolomic studies have shown that *Salmonella*-infected mice exhibit a sharp decline in intestinal propionate, effectively releasing the “brake” on bacterial invasion while weakening the host’s ability to control systemic inflammation, thereby facilitating pathogen dissemination beyond the gut [55,56].

Butyrate serves as the principal energy source for colonic epithelial cells and is indispensable for maintaining epithelial barrier integrity, promoting TJ protein synthesis, and inducing regulatory T cell (Treg) differentiation. The inflammatory environment triggered by *Salmonella* exerts a devastating impact on butyrate-producing bacteria [57]. ROS and nitrate generated during inflammation are highly toxic to these obligate anaerobes, while host-derived antimicrobial peptides (e.g. RegIIIγ) may inadvertently target these beneficial microbes. It has been reported that *Salmonella* infection significantly reduces cecal butyrate concentrations in mouse models, and its depletion leads to epithelial barrier breakdown and increased intestinal permeability [58]. Moreover, as a histone deacetylase inhibitor, butyrate is essential for promoting Treg differentiation and the production of anti-inflammatory cytokines such as IL-10. Therefore, butyrate deficiency impairs these immunoregulatory processes, whereas butyrate supplementation enhances host resistance and alleviates inflammation-associated tissue damage [59].

### Changes in amino acid derivatives aggravate gut inflammation

Following *Salmonella* invasion, host amino acid metabolic networks – particularly within the intestine – undergo profound reprogramming, with alterations in tryptophan metabolism being especially prominent (Figure 3(B)) [60]. As an essential amino acid, tryptophan metabolism spans both the intestine and liver and extends beyond protein synthesis and serotonin production to encompass immune regulation, barrier maintenance, and neurophysiological processes, all of which are critical for homeostasis [61]. Under conditions of dysbiosis, *Salmonella* infection reshapes tryptophan metabolism primarily through three major pathways. The first is the kynurenine pathway. Infection-induced inflammation, especially signaling mediated by IFN-γ and IFN-1, drives the upregulation of indoleamine 2,3-dioxygenase 1 (IDO1) in intestinal epithelial and immune cells [62]. As the rate-limiting enzyme of this pathway, IDO1 catalyzes the conversion of tryptophan into N-formylkynurenine, which is subsequently metabolized into kynurenine, 3-hydroxykynurenine, quinolinic acid, and related metabolites, ultimately contributing to NAD^+^ biosynthesis. However, under persistent inflammatory conditions, kynurenine pathway metabolites – particularly kynurenine itself – can exert immunosuppressive effects, impair pathogen clearance, and are closely associated with the development of chronic infection [63]. The second pathway is the microbial indole pathway, largely governed by commensal microbiota. Under physiological conditions, gut bacteria such as *Escherichia coli* convert tryptophan into a variety of indole derivatives, including indole, indole-3-propionic acid, indole-3-acetic acid, and indole acrylic acid [64]. Several of these metabolites (e.g. indole-3-propionic acid and indole-3-aldehyde) act as potent ligands for the aryl hydrocarbon receptor (AhR). Activation of AhR supports epithelial barrier integrity by promoting mucus secretion and TJ protein expression. It also regulates the function of group 3 innate lymphoid cells (ILC3s) and Th17 cells, enhancing IL-22 production, which is essential for host defense and tissue repair [65]. In an in vivo mouse model, inflammation and tryptophan depletion induced by *Salmonella* markedly suppress indole-producing commensals, leading to reduced levels of beneficial indole derivatives and diminished AhR signaling. This, in turn, weakens barrier function and paradoxically facilitates *Salmonella* invasion and colonization [66]. The third pathway is serotonin (5-hydroxytryptamine, 5-HT) synthesis, which primarily occurs in intestinal enterochromaffin cells. The effects of *Salmonella* infection on this pathway appear paradoxical. On the one hand, IDO1-mediated tryptophan depletion may limit substrate availability for serotonin synthesis; on the other hand, infection-associated inflammatory cytokines, such as IFN-γ, can directly upregulate tryptophan hydroxylase 1 (TPH1), potentially serving as a compensatory mechanism [67]. Overall, the net contribution of this pathway during *Salmonella* infection remains incompletely understood but is generally considered less dominant than the kynurenine and indole pathways.

Beyond tryptophan, the metabolism of other amino acids is also markedly altered during infection. In the gut, tyrosine can be metabolized by specific anaerobic bacteria through aromatic amino acid fermentation pathways to generate phenolic metabolites, including p-cresol and 4-ethylphenol. Under normal physiological conditions, these metabolites are maintained at relatively low levels and can be further detoxified and eliminated through host-mediated conjugation reactions, such as sulfation and glucuronidation. However, gut microbiota dysbiosis induced by pathogenic infections may alter the abundance and metabolic activity of tyrosine-utilizing bacteria, resulting in excessive production and accumulation of phenolic metabolites [68]. Studies have shown that high concentrations of p-cresol can induce oxidative stress, impair mitochondrial function, and disrupt epithelial energy metabolism, thereby reducing epithelial cell viability and compromising intestinal barrier integrity [69]. In addition, p-cresol can inhibit Na^+^/K^+^-ATPase activity, leading to impaired ion transport and disruption of epithelial electrolyte homeostasis, which further increases intestinal permeability, promotes microbial translocation, and exacerbates inflammatory responses. Moreover, excessive accumulation of p-cresol and related metabolites may activate NF-κB signaling through oxidative stress-mediated mechanisms, thereby promoting the production of pro-inflammatory cytokines and amplifying inflammatory responses [70,71]. Although direct evidence demonstrating that *Salmonella* infection inevitably increases intestinal levels of p-cresol and 4-ethylphenol remains limited, *Salmonella*-induced microbiota disruption and metabolic remodeling may create favorable conditions for the accumulation of these potentially harmful metabolites. Therefore, alterations in tyrosine-derived phenolic metabolism may represent an additional pathway through which *Salmonella*-associated dysbiosis contributes to intestinal barrier impairment and inflammatory injury.

Branched-chain amino acids (BCAAs), including leucine, isoleucine, and valine, are not only essential substrates for protein synthesis but also serve as critical signaling molecules involved in the regulation of cellular metabolism and immune responses. Among them, leucine is a potent nutrient signal that activates mechanistic target of rapamycin complex 1 (mTORC1) through the Sestrin2–GATOR–Rag GTPase pathway. Activated mTORC1 subsequently regulates the phosphorylation of downstream effectors, including ribosomal protein S6 kinase 1 (S6K1) and eukaryotic initiation factor 4E-binding protein 1 (4E-BP1), thereby promoting protein synthesis, epithelial cell proliferation, and tissue repair to maintain intestinal barrier homeostasis [72–74]. During *Salmonella* infection, microbiota dysbiosis and inflammatory responses can disrupt BCAA metabolism and result in dysregulated mTORC1 signaling. Reduced BCAA availability may decrease mTORC1 activity, impair intestinal epithelial proliferation, and downregulate TJ proteins, including ZO-1, occludin, and claudins, thereby compromising barrier repair and facilitating *Salmonella* colonization. Conversely, persistent dysregulation of BCAA signaling may lead to excessive mTORC1 activation, enhancing pro-inflammatory immune responses and exacerbating intestinal inflammation [75,76]. Therefore, the BCAA–mTORC1 axis may represent an important regulatory pathway linking *Salmonella*-induced metabolic disturbances, intestinal barrier disruption, and inflammatory injury.

### Dysregulation of bile acids promotes intestinal inflammation

Bile acids not only play essential roles in lipid digestion and absorption but also function as key signaling molecules. By activating the farnesoid X receptor (FXR) and the G protein–coupled bile acid receptor 1 (TGR5), they broadly regulate metabolic processes, inflammatory responses, and the maintenance of intestinal barrier function (Figure 3(C)). Bacterial infection can profoundly disrupt both the composition of the bile acid pool and its associated signaling networks [77].

Primary bile acids, including cholic acid (CA) and chenodeoxycholic acid (CDCA), as well as their glycine- or taurine-conjugated forms, are synthesized in the liver and secreted into the intestine via bile [78]. Upon reaching the ileum, bile acids activate FXR signaling in intestinal epithelial cells, inducing fibroblast growth factor 15/19 (FGF15/19) expression and promoting antimicrobial peptide production, thereby maintaining epithelial barrier integrity and limiting pathogen expansion [79,80]. During *Salmonella* infection, systemic inflammation and host stress responses may disrupt hepatic bile acid synthesis and transport, while microbiota dysbiosis may impair the ileal FXR–FGF15/19 feedback loop, ultimately altering the bile acid pool composition [81].

In the gut, conjugated bile acids are deconjugated by microbial bile salt hydrolases (BSHs) and subsequently converted into secondary bile acids, such as deoxycholic acid (DCA) and lithocholic acid (LCA), through 7α-dehydroxylation mediated by specific anaerobic bacteria, particularly members of the Clostridium genus within the Firmicutes phylum [82]. *Salmonella*-induced dysbiosis, especially the depletion of secondary bile acid-producing bacteria, generally reduces the levels of DCA and LCA. However, the effects of secondary bile acids are highly context- and dose-dependent. Physiological levels of LCA can activate TGR5 and promote anti-inflammatory signaling, whereas excessive DCA accumulation may induce oxidative stress, enhance inflammatory responses, and impair epithelial integrity [83,84]. Therefore, bile acid dysregulation reflects not simply a reduction in specific metabolites but rather an imbalance between diminished protective signaling and the accumulation of potentially harmful inflammatory mediators.

Importantly, *Salmonella* can also sense and respond to bile acid signals, using them as environmental cues to regulate intestinal adaptation and virulence expression [85]. Bile acids modulate *Salmonella* pathogenicity by influencing virulence regulatory networks. For example, intestinal bile acid conditions suppress the expression of SPI-1-associated invasion genes encoding the T3SS, thereby preventing excessive epithelial invasion during luminal colonization. After internalization into host cells, bile acid-mediated signaling can also influence SPI-2-associated regulatory pathways, facilitating intracellular adaptation and bacterial persistence [86]. Thus, bile acids function not only as host-derived antimicrobial signals but also as ecological cues shaping *Salmonella* colonization and virulence strategies.

Overall, *Salmonella* infection is accompanied by profound alterations in bile acid metabolism, characterized by reduced secondary bile acid production, increased proportions of primary and conjugated bile acids, and disrupted FXR/TGR5 signaling. Impaired FXR activation may decrease antimicrobial peptide expression, such as α-defensins, weakening innate intestinal defense, whereas abnormal TGR5 signaling may affect GLP-1 secretion, energy metabolism, and macrophage function, thereby exacerbating metabolic and immune dysregulation [87]. Collectively, bile acid imbalance represents an important metabolic link connecting *Salmonella*-induced microbiota disruption, impaired host immunity, and reduced CR.

## *Salmonella*-induced dysbiosis triggers inflammatory responses, which play a crucial role in disrupting intestinal CR

### The manner in which intestinal dysbiosis contributes to the onset of IBD

Microbial metabolites play a pivotal role in the initiation and progression of IBD (Figure 4(A)). Dysbiosis leads to a progressive depletion of protective metabolites alongside the accumulation of deleterious compounds, thereby directly triggering inflammatory responses [88]. Among these, SCFAs and their derivatives, tryptophan metabolites, and polyamines are key regulators of intestinal barrier integrity and immune homeostasis. They contribute to inflammation control by promotingTreg differentiation, enhancing barrier function, and suppressing pro-inflammatory cytokine production [89]. Notably, butyrate serves as an indispensable energy source for colonic epithelial cells. It upregulates the expression of TJ proteins and reduces intestinal permeability, thereby maintaining mucosal integrity [90]. However, during dysbiosis, the balance of SCFA production is disrupted. For instance, a decline in butyrate-producing bacteria, such as *Faecalibacterium prausnitzii* and *Roseburia* spp., leads to reduced SCFA levels, ultimately impairing barrier function and exacerbating inflammation [91]. Concurrently, the expansion of pathogenic bacteria and their aberrant antigenic components increases the likelihood of recognition by pattern recognition receptors (PRRs), including Toll-like receptors (TLRs) and NOD-like receptors (NLRs). In patients with IBD, these receptors often exhibit genetic polymorphisms that compromise their capacity for effective detection and clearance [92]. This “double hit” – the loss of beneficial signals coupled with the amplification of harmful stimuli – drives the intestinal immune system into a state of persistent false alarm. As a result, excessive production of pro-inflammatory cytokines ensues, leading to sustained immune-mediated damage to intestinal tissues and perpetuation of inflammation [93].

**Figure 4.**
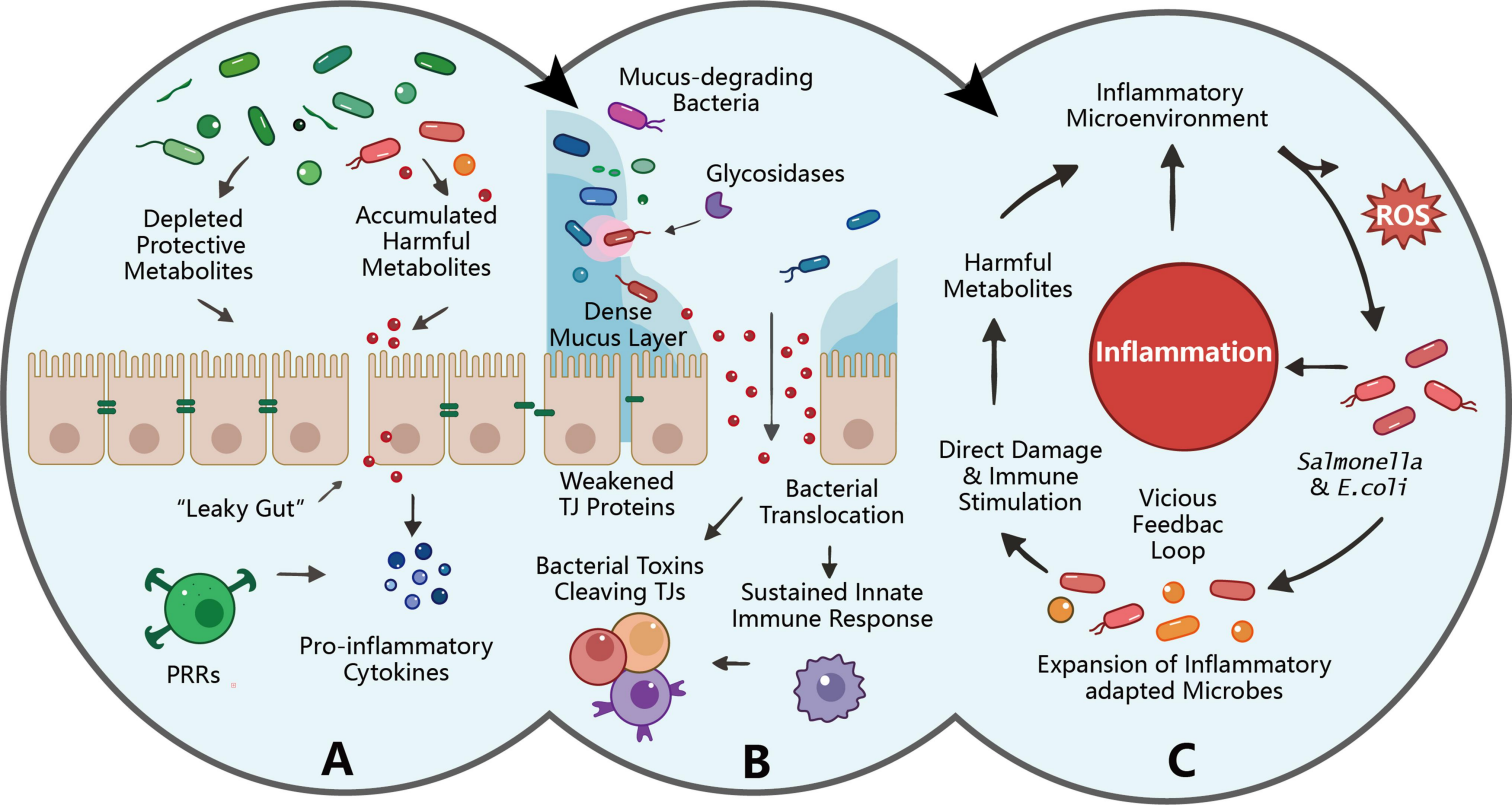
Schematic diagram of the mechanisms underlying gut dysbiosis-induced IBD. (A) Metabolite imbalance and immune misactivation. Dysbiosis reduces protective metabolites and accumulates harmful ones, increasing intestinal permeability. (B) Barrier dysfunction and bacterial translocation. Enrichment of mucus-degrading bacteria and reduced butyrate disrupt TJs, impairing barrier integrity and promoting bacterial translocation, which sustains innate immune activation. (C) Inflammation-dysbiosis vicious cycle. Inflammatory microenvironmental changes (e.g. increased ROS) favor expansion of inflammation-adapted microbes (e.g. *Salmonella, E. coli*).

Furthermore, dysbiotic microbial communities synergistically compromise both the structural and functional integrity of the intestinal barrier, thereby facilitating the propagation of inflammation (Figure 4(B)). In a healthy state, the intestinal barrier consists of tightly connected epithelial cells overlaid by a dense mucus layer, which effectively segregates the vast population of luminal microorganisms from host tissues [94]. Dysbiosis disrupts this defense on multiple fronts.

On the one hand, certain pathogenic taxa, such as mucus-degrading bacteria, secrete specific glycosidases that actively cleave the glycoprotein scaffold of the mucus layer, leading to its thinning or even focal depletion [95,96]. Consistently, studies in antibiotic-treated and inflammatory mouse models have shown that expansion of mucin-degrading bacteria is accompanied by reduced MUC2 expression, decreased mucus layer thickness, and increased bacterial proximity to the epithelial surface, confirming their contribution to mucus barrier disruption [97].

On the other hand, the reduction in butyrate availability following microbial imbalance directly impairs epithelial energy metabolism and gene expression, weakening TJ architecture and promoting the development of increased intestinal permeability (“leaky gut”) [98–100]. Experimental evidence from germ-free and antibiotic-depleted mouse models demonstrates that depletion of butyrate-producing bacteria decreases epithelial expression of TJs, including ZO-1, occludin, and claudin-1, whereas butyrate supplementation restores barrier integrity and reduces FITC-dextran permeability [101].

In addition, toxins produced by opportunistic pathogens can directly cleave or modify TJs, further exacerbating mucosal damage. For example, animal studies have demonstrated that *Clostridioides difficile* toxins TcdA and TcdB disrupt epithelial TJs through Rho GTPase inactivation, resulting in increased intestinal permeability and inflammatory tissue injury [102]. Similarly, *S*. Typhimurium infection models have shown that T3SS-secreted effectors, including SopE and SopB, induce cytoskeletal rearrangement and redistribution of TJ proteins, facilitating bacterial translocation across the epithelial barrier [103]. The resulting loss of barrier function permits the translocation of bacterial antigens and their metabolites from the lumen into the submucosa, where they come into direct contact with immune cells and continuously provoke robust innate immune responses.

More importantly, a self-reinforcing vicious cycle emerges between microbial dysbiosis and intestinal inflammation that is difficult to disrupt [104]. Initial dysbiosis triggers immune activation and barrier impairment, thereby promoting the onset of inflammation (Figure 4(C)). Once established, inflammation profoundly alters the intestinal milieu, including increased oxygen availability at inflamed sites and changes in local chemical composition due to exudation [105]. This inflammatory microenvironment is highly selective: it suppresses the growth of obligate anaerobic commensals while favoring the expansion of oxygen-tolerant, pro-inflammatory microorganisms, such as *Enterococcus* spp. and certain *Escherichia coli* strains. Consequently, a microbial community adapted to inflammatory conditions becomes established [106]. Evidence indicates that activated immune cells, particularly neutrophils, generate large amounts of ROS during inflammation [107]. These ROS can oxidize relatively inert thiosulfate into tetrathionate within the gut. While most commensal anaerobes are unable to utilize tetrathionate, *Salmonella* and some members of the phylum Proteobacteria harbor the *ttr* gene cluster, which encodes a respiratory pathway using tetrathionate as a terminal electron acceptor. This mode of respiration yields more ATP than fermentation, conferring a marked growth advantage to these bacteria [108].

At the same time, inflammation-induced damage to the mucus layer and epithelial cells releases sialic acid residues from mucins. Certain *Salmonella* species and *Clostridioides difficile* possess the *nan* operon, enabling them to preferentially utilize sialic acid as a carbon source. This metabolic capability provides a competitive edge over commensals that rely primarily on dietary fiber. As a result, the proliferation of these organisms displaces obligate anaerobic symbionts, further exacerbating dysbiosis [109]. Additionally, dysbiotic communities can amplify inflammation through alternative metabolic pathways. For instance, excessive production of cytotoxic hydrogen sulfide by sulfate-reducing bacteria, or dysregulated bile acid metabolism leading to increased levels of secondary bile acids, can directly damage epithelial tissues and stimulate immune responses. These processes further intensify and stabilize the dysbiotic state, perpetuating the inflammatory cycle [110,111].

### Molecular mechanisms of *Salmonella*-induced IBD via gut dysbiosis

*Salmonella* infection–induced dysbiosis that subsequently exacerbates inflammatory enteritis represents a dynamic process involving intricate interactions among the pathogen, host immunity, and the intestinal microbiota. At the molecular level, this process centers on the recognition of pathogen-associated molecular patterns (PAMPs) and danger-associated molecular patterns (DAMPs) by cytosolic PRRs, followed by the activation of multiple inflammatory signaling cascades. Among these, the NLRP3 inflammasome pathway, the NAIP/NLRC4 inflammasome pathway, and the non-canonical inflammasome pathway mediated by Caspase-11 play pivotal roles, collectively forming a central hub through which *Salmonella* disrupts intestinal homeostasis and drives inflammatory cascades [112–114].

As an invasive enteric pathogen, *Salmonella* initiates infection by deploying its T3SS to inject a repertoire of effector proteins into the host cell cytosol, thereby directly triggering the assembly and activation of the NAIP/NLRC4 inflammasome. Members of the NAIP family function as intracellular sensors of bacterial ligands, specifically recognizing T3SS structural components such as the needle protein (e.g. PrgJ) or rod proteins (e.g. FliC protein) [115]. Upon entry of these components into the cytosol, specific NAIP molecules – such as NAIP5/6 in mice, which detect needle/rod proteins, and NAIP2, which recognizes flagellin – bind their ligands and subsequently recruit and activate the adaptor protein NLRC4 (Figure 5(A)). Activated NLRC4 undergoes oligomerization to form a platform that recruits pro-caspase-1, which is then activated through autocleavage. Activated Caspase-1 exerts two essential functions. First, it processes the precursor forms of pro-inflammatory cytokines, pro-IL-1β and pro-IL-18, into their mature, bioactive forms, which are subsequently released extracellularly. Second, it cleaves gasdermin D (GSDMD), whose N-terminal fragment (GSDMD-N) forms pores in the plasma membrane and, together with the activation of NINJ1, drives cell lysis, culminating in pyroptotic inflammatory cell death [116]. Within the intestinal environment, activation of the NAIP/NLRC4 inflammasome exhibits dual effects. On the one hand, the release of IL-1β and IL-18 potently recruits neutrophils and other immune cells, thereby amplifying local inflammatory responses [117]. On the other hand, pyroptosis of infected epithelial and immune cells, while contributing to the elimination of intracellular bacteria, simultaneously compromises the integrity of the intestinal epithelial barrier [118]. This barrier disruption facilitates the translocation of commensal microbes and their products (e.g. LPS and microbial DNA) from the lumen into the submucosa, thereby promoting dysbiosis and subsequent immune activation, ultimately creating a permissive environment for the development of chronic inflammation.

**Figure 5.**
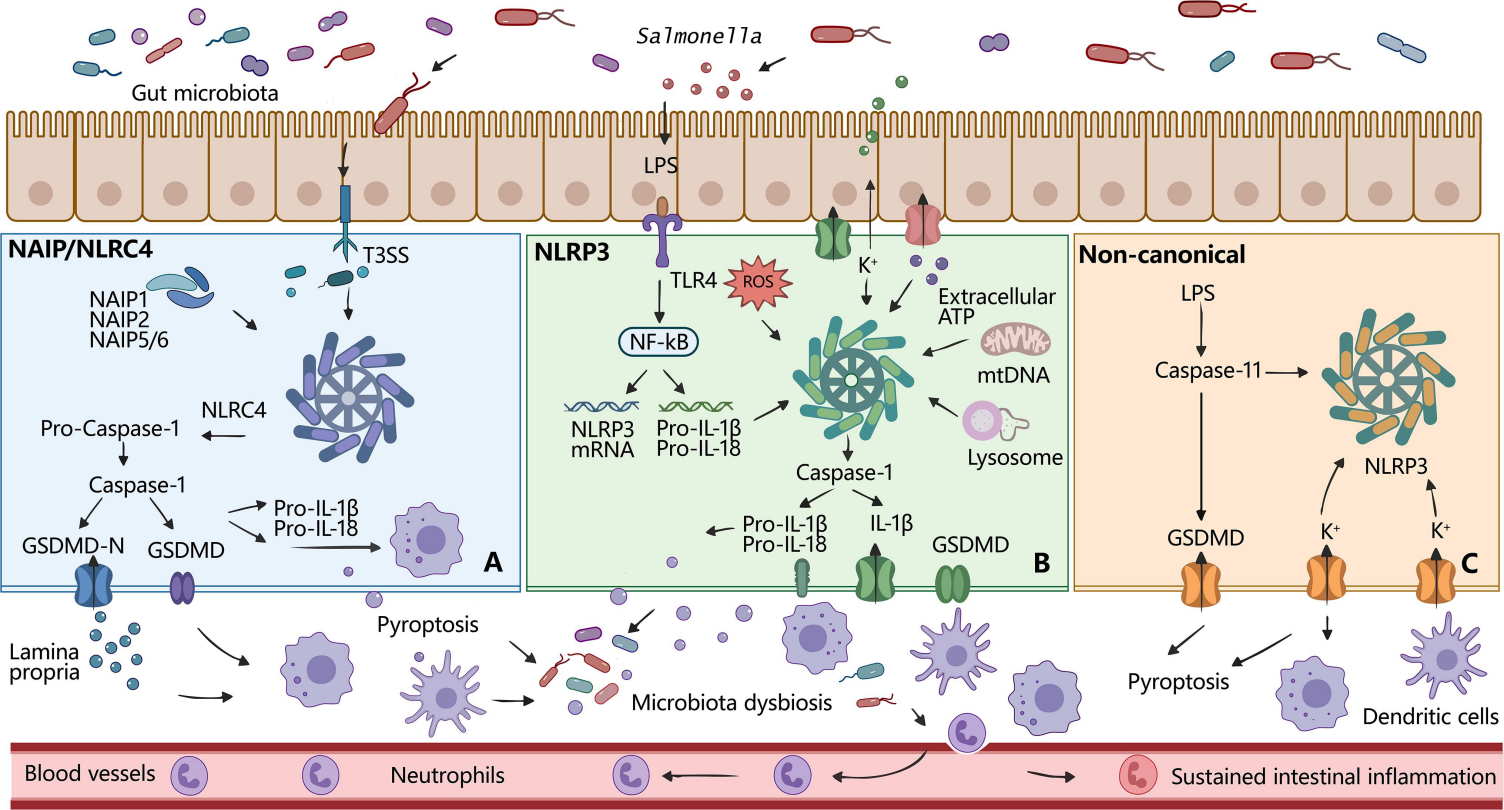
Schematic diagram of *Salmonella*-induced intestinal inflammation via inflammasome activation and pyroptosis. (A) Inflammasome activation pathways. *Salmonella* activates NAIP/NLRC4 pathway via T3SS and flagellin (FliC, PrgJ). (B) Extracellular *Salmonella* LPS triggers TLR4/NF-κB priming, while cytosolic ATP, ROS, and lysosomal cathepsin leakage activates NLRP3-caspase-1, and caspases-1 cleave pro-IL-1β and pro-IL-18 into mature IL-1β and IL-18, and cleave GSDMD. The GSDMD-N forms membrane pores, inducing pyroptosis. Mitochondrial damage releases mtDNA, amplifying inflammation. (C) Cytosolic LPS activates non-canonical caspase-11. These pathways converge on caspase-1/11 activation. A decrease in intracellular potassium ion concentration is also a key factor in activating the NLRP3 inflammasome.

*Salmonella* infection, together with the dysbiosis and cellular injury it induces, provides multiple converging signals for the activation of the NLRP3 inflammasome (Figure 5(B)) [119]. The NLRP3 inflammasome is a multiprotein complex composed of the sensor molecule NLRP3, the adaptor protein ASC, and the effector protease Caspase-1, and it plays a central role in innate immune responses [120]. Its activation generally follows a two-step process: priming and activation. The priming signal is primarily initiated by Toll-like receptor–mediated recognition of microbial components such as LPS, which activates the NF-κB pathway and upregulates the transcription of NLRP3, pro-IL-1β, and pro-IL-18. LPS derived from *Salmonella* itself, as well as that from translocated commensal bacteria due to barrier disruption, can both provide potent priming signals through TLR4 [121]. In contrast, the activation signal arises from a broader range of stimuli. *Salmonella* invasion induces multiple forms of cellular stress, including the generation of ROS by bacterial effectors or host antimicrobial responses, potassium efflux triggered by bacterial toxins, lysosomal damage, and the release of DAMPs such as extracellular ATP and mitochondrial DNA from injured or dying cells [122]. The convergence of these signals promotes conformational changes in NLRP3, enabling its assembly with ASC, which in turn recruits pro-caspase-1. Following autocleavage, active Caspase-1 mediates the maturation of IL-1β and IL-18 and cleaves GSDMD, thereby inducing pyroptosis, analogous to the downstream effects observed with NLRC4 activation. In the context of bacterial infection, the NLRP3 inflammasome often functions in coordination or sequence with the NAIP/NLRC4 inflammasome [123]. During the early phase of infection, NAIP/NLRC4 rapidly responds to directly invading bacterial components. As infection progresses and is accompanied by barrier disruption and microbial translocation, NLRP3 activation – driven by DAMPs and sustained microbial signals – becomes increasingly prominent. This sustained activation amplifies inflammatory responses and regulates the effector functions of multiple immune cell populations, including macrophages and dendritic cells in the intestinal lamina propria, thereby playing a crucial role in both acute and chronic *Salmonella*-induced intestinal inflammation [124].

In addition to the canonical Caspase-1–dependent inflammasome pathways described above, *Salmonella* infection robustly activates the non-canonical inflammasome pathway mediated by Caspase-11 (with human orthologs Caspase-4/5) [125]. This pathway functions through the direct recognition of cytosolic LPS and constitutes a specialized host defense mechanism against Gram-negative bacterial infections. When *Salmonella* replicates within the cytosol or is internalized by host cells, its outer membrane component, LPS, can gain access to the cytoplasm via bacterial lysis or specific transport mechanisms. Caspase-11 directly binds the lipid A moiety of LPS via its C-terminal domain, leading to its oligomerization and activation [126]. Unlike Caspase-1, activated Caspase-11 does not directly process pro-IL-1β or pro-IL-18. Instead, it specifically cleaves GSDMD, thereby inducing pyroptosis (Figure 5(C)). The formation of membrane pores during pyroptosis not only facilitates the elimination of infected cells but also promotes the release of intracellular contents, including large amounts of DAMPs and potassium ions. A reduction in intracellular potassium concentration has been established as a key trigger for NLRP3 inflammasome activation [127]. Consequently, the Caspase-11 pathway amplifies inflammation by inducing pyroptosis and promoting secondary activation of NLRP3, thereby forming a potent feed-forward inflammatory loop. In the context of intestinal *Salmonella* infection, this amplification circuit is particularly critical. First, it provides an additional layer of host defense by directly sensing cytosolic LPS. Second, Caspase-11–mediated pyroptosis exacerbates the loss of epithelial and immune cells, thereby worsening intestinal barrier dysfunction. More importantly, by activating NLRP3, this pathway tightly couples the non-canonical and canonical inflammasome pathways, leading to a robust surge in IL-1β and IL-18 production and markedly intensifying the inflammatory response [128]. Experimental studies have demonstrated that Caspase-11–deficient mice exhibit significantly attenuated intestinal inflammation and reduced pathological damage following *Salmonella* infection, underscoring the central role of this pathway in driving *Salmonella*-associated enteritis [129,130]. Collectively, these findings highlight the importance of maintaining intestinal microbiota homeostasis as a critical defense against *Salmonella* infection. Beyond directly triggering immune responses via its T3SS, *Salmonella* further amplifies host inflammation by disrupting the intestinal barrier and promoting dysbiosis. Future therapeutic strategies may therefore focus on restoring microbial balance or supplementing beneficial bacterial strains to reinforce barrier integrity, prevent dysbiosis, and ultimately mitigate *Salmonella*-induced IBD.

## Summary and future prospects

This review centers on the “gut microbiota–inflammatory pathways–intestinal injury” axis following infection with S. Typhimurium. It highlights how *Salmonella* actively induces gut dysbiosis – characterized by a reduction in SCFA-producing bacteria and decreased microbial diversity – through multiple strategies, including nutrient competition, secretion of antimicrobial factors, and manipulation of host inflammatory responses. Two principal mechanisms by which *Salmonella* disrupts CR are summarized. On the one hand, *Salmonella* fundamentally alters the composition of microbiota-derived metabolites, thereby undermining CR across multiple dimensions, including energy supply, epithelial barrier integrity, and immune regulation [131]. On the other hand, dysbiosis reshapes the local immune microenvironment, leading to aberrant activation and amplification of the inflammasome pathway centered on NLRP3 and Caspase-1. This process drives excessive pyroptosis and a surge of IL-1β and IL-18, ultimately contributing to severe inflammatory colitis and increasing the risk of systemic infection [132].

At the mechanistic level, NLRP3 was traditionally considered to be primarily activated by PAMPs. However, emerging evidence suggests that it functions more fundamentally as a sensor of metabolic stress, with microbiota-derived metabolites serving as key modulators. Studies have shown that specific microbial metabolites can directly or indirectly trigger NLRP3 activation. For instance, secondary bile acids, such as lithocholic acid, at high concentrations can activate NLRP3 by compromising lysosomal integrity [133,134], while trimethylamine N-oxide (TMAO) promotes microtubule acetylation, thereby providing a structural platform for NLRP3 assembly [135]. Conversely, microbial metabolites can also regulate NLRP3 expression and activity. For example, primary bile acids, such as CDCA, activate the TGR5 receptor and subsequently the cAMP–PKA signaling pathway, leading to direct phosphorylation of NLRP3 and inhibition of its oligomerization [136]. Despite these advances, the crosstalk between the NLRP3 inflammasome and microbiota-driven metabolic reprogramming remains insufficiently understood. Future studies integrating spatial multi-omics approaches, gut-restricted therapeutics, and engineered bacteria-mediated metabolic remodeling may enable “region-specific” precision control of NLRP3 activity – preserving barrier integrity on the luminal side while suppressing aberrant inflammation at the mucosal interface – thereby offering novel therapeutic strategies for IBD.

Based on these mechanisms, functional probiotics can be rationally screened for targeted applications, including strains capable of suppressing NLRP3 inflammasome assembly or inhibiting Caspase-1 activation. Previous studies have demonstrated that *Lactobacillus johnsonii* markedly attenuates the activation of the TLR4/NF-κB/NLRP3 signaling pathway in *Salmonella*-challenged IPEC-J2 cell models, thereby reducing the expression of pro-inflammatory cytokines and preserving epithelial TJ integrity [137]. Furthermore, these mechanistic insights provide a foundation for identifying natural antimicrobial agents with dual functions of inflammation suppression and metabolic regulation. For example, berberine has been shown to inhibit pro-inflammatory signaling pathways, including TLR4/NF-κB and MAPK pathways, thereby reducing the production of inflammatory cytokines such as IL-1β, IL-6, and TNF-α and limiting leukocyte recruitment. In a rat model of IBD, berberine modulated tryptophan metabolism, enhanced gut microbial diversity, and promoted the proliferation of beneficial bacteria, including *Lactobacillus*, *Clostridium*, and *Akkermansia* [138]. The two aforementioned approaches can provide a theoretical basis for the research and development of novel microecological preparations aimed at promoting human health. In addition, bacteriophages have been extensively investigated as targeted antimicrobial agents against *Salmonella* infections, with studies showing that phage administration can effectively reduce *Salmonella* colonization in the gastrointestinal tract of broiler chickens [139]. This approach demonstrates favorable potential and ample room for development within the livestock farming sector. A comprehensive understanding of the integrated mechanisms by which *Salmonella* disrupts CR through microbiota dysbiosis, metabolic reprogramming, and inflammasome activation will provide a theoretical basis for developing microbiota-based intervention strategies involving probiotics, natural antimicrobial compounds, and bacteriophages. Such insights may also facilitate the development of novel adjunctive approaches for the prevention and treatment of *Salmonella* infections and associated inflammatory intestinal disorders.

## Data sharing statement

Data sharing does not apply to this article, as no new data were created or analyzed in this study.

## References

[1] Yang J, Xiao Y, Cui J. A 4-guanidinobutanoic acid-SLC36A1 axis drives a microbiota‒host feedback loop to regulate intestinal homeostasis. Gut Microbes. 2026;18(1):2639216. doi: 10.1080/19490976.2026.2639216 PMC12969751 41782409

[2] Fu Y, Lyu J, Wang S. The role of intestinal microbes on intestinal barrier function and host immunity from a metabolite perspective. Front Immunol. 2023;14:1277102. doi: 10.3389/fimmu.2023.1277102 PMC10591221 37876938

[3] Cadwell K, Loke P. Gene–environment interactions shape the host–microbial interface in inflammatory bowel disease. Nat Immunol. 2025;26(7):1023–17. doi: 10.1038/s41590-025-02197-5 PMC12443487 40528029

[4] Krishnan M.P.S, Goyal B, Nampui L, Gupta S.C. The role of microbiome in gastrointestinal cancer. Int Rev Cell Mol Biol. 2025;395:67–98. doi:10.1016/bs.ircmb.2024.12.009 40653356

[5] Li N, Guo X. The gut microbiota and host immunity synergistically orchestrate colonization resistance. Gut Microbes. 2026;18(1):2611545. doi: 10.1080/19490976.2025.2611545 PMC12773493 41482864

[6] Furuichi M, Kawaguchi T, Pust M-M. Commensal consortia decolonize Enterobacteriaceae via ecological control. Nature. 2024;633(8031):878–886. doi: 10.1038/s41586-024-07960-6 PMC11424487 39294375

[7] Jacobs SO, Muenchau S, Uckeley ZM. Hypoxia increases susceptibility of human intestinal epithelial cells to rotavirus infection through repression of interferon induction. Gut Microbes. 2025;17(1):2560593. doi: 10.1080/19490976.2025.2560593 PMC12456215 40977474

[8] Zhang Y, Si L, Shu X. Gut microbiota contributes to protection against porcine deltacoronavirus infection in piglets by modulating intestinal barrier and microbiome. Microbiome. 2025;13(1):93. doi: 10.1186/s40168-025-02092-z PMC11974153 40189556

[9] Gul E, Enz U, Maurer L. Intraluminal neutrophils limit epithelium damage by reducing pathogen assault on intestinal epithelial cells during Salmonella gut infection. PLOS Pathog. 2023;19(6):e1011235. doi: 10.1371/journal.ppat.1011235 PMC10337893 37384776

[10] Chen H, Shang K, Jia Y. *Salmonella enterica* serovar Typhimurium uses T6SS to promote bacterial adaptability and enhance its survival of niches in broiler chickens. Poult Sci. 2025;104(8):105277. doi: 10.1016/j.psj.2025.105277 PMC12151195 40412062

[11] Chen S, Du F, Shang K. Colonization mediated by T6SS-ClpV disrupts host gut microbiota and enhances virulence of *Salmonella enterica* serovar Typhimurium. J Agric Food Chem. 2024;72(34):19155–19166. doi: 10.1021/acs.jafc.4c03735 39161106

[12] Ma C, Zou Z, Zhao W. The gut microbiota in Salmonella typhi infection and translocation: mechanisms of colonization resistance, pathogen subversion, and prospects for microecological intervention. Microb Pathog. 2026;213:108381. doi: 10.1016/j.micpath.2026.108381 41690650

[13] Li Y, Xu Y, Jin C. Salmonella-NLRP3 inflammasome crosstalk: Host defense activation versus bacterial immune evasion strategies. JIR. 2025;18:5133–5148. doi: 10.2147/JIR.S519902 PMC12009050 40255664

[14] Kandari A, Odat MA, Alzaid F. Biotics and bacterial function: impact on gut and host health. Isme J. 2024;18(1). doi: 10.1093/ismejo/wrae226 PMC11631128 39499657

[15] Rinninella E, Raoul P, Cintoni M. What is the healthy gut microbiota composition? A changing ecosystem across age, environment, diet, and diseases. Microorganisms. 2019;7(1):14. doi: 10.3390/microorganisms7010014 PMC6351938 30634578

[16] Gómez-Duarte OG, Ogra PL. Editorial: impact of microbiome on gut mucosal immunity in health and disease. Front Immunol. 2022;13:1005156. doi: 10.3389/fimmu.2022.1005156 PMC9532250 36211406

[17] Gomaa EZ. Human gut microbiota/microbiome in health and diseases: a review. Antonie Van Leeuwenhoek. 2020;113(12):2019–2040. doi: 10.1007/s10482-020-01474-7 33136284

[18] Thursby E, Juge N. Introduction to the human gut microbiota. Biochem J. 2017;474(11):1823–1836. doi: 10.1042/BCJ20160510 PMC5433529 28512250

[19] Crouch LI, Rodrigues CS, Bakshani CR. The role of glycans in health and disease: regulators of the interaction between gut microbiota and host immune system. Semin Immunol. 2024;73:101891. doi: 10.1016/j.smim.2024.101891 39388764

[20] Rowland I, Gibson G, Heinken A. Gut microbiota functions: metabolism of nutrients and other food components. Eur J Nutr. 2018;57(1):1–24. doi: 10.1007/s00394-017-1445-8 PMC5847071 28393285

[21] Goethel A, Croitoru K, Philpott DJ. The interplay between microbes and the immune response in inflammatory bowel disease. J Physiol. 2018;596(17):3869–3882. doi: 10.1113/JP275396 PMC6117586 29806140

[22] Santilli A, Stefanopoulos S, Cresci GAM. The gut barrier and chronic diseases. Curr Opin Clin Nutr Metab Care. 2022;25(3):178–185. doi: 10.1097/MCO.0000000000000820 PMC9246825 35125386

[23] Palm NW, de Zoete MR, Flavell RA. Immune–microbiota interactions in health and disease. Clin Immunol. 2015;159(2):122–127. doi: 10.1016/j.clim.2015.05.014 PMC4943041 26141651

[24] Zilberman-Schapira G, Zmora N, Itav S. The gut microbiome in human immunodeficiency virus infection. BMC Med. 2016;14(1):83. doi: 10.1186/s12916-016-0625-3 PMC4891875 27256449

[25] Cryan JF, O’Riordan KJ, Cowan CSM. The microbiota-gut-brain axis. Physiol Rev. 2019;99(4):1877–2013. doi: 10.1152/physrev.00018.2018 31460832

[26] Dalile B, Van Oudenhove L, Vervliet B. The role of short-chain fatty acids in microbiota–gut–brain communication. Nat Rev Gastroenterol Hepatol. 2019;16(8):461–478. doi: 10.1038/s41575-019-0157-3 31123355

[27] Mayer EA. Gut feelings: the emerging biology of gut–brain communication. Nat Rev Neurosci. 2011;12(8):453–466. doi: 10.1038/nrn3071 PMC3845678 21750565

[28] Blondel CJ, Amaya FA, Reyes-Méndez F. Deciphering the Salmonella T6SS toolkit: two decades of research decoding a versatile bacterial weapon. J Bacteriol. 2025;207(7):e0018825. doi: 10.1128/jb.00188-25 PMC12288458 40511914

[29] Liu C, Dai J, Liao C. From past to present: the crosstalk between type VI secretion system of Salmonella and host. Arch Microbiol. 2025;208(1):56. doi: 10.1007/s00203-025-04608-5 41364199

[30] Parra-Calisto A, Santiviago CA, Blondel CJ. Type VI secretion systems in Salmonella encode New effectors with putative antibacterial and anti-Eukaryotic activities. Microorganisms. 2026;14(6):1232. doi: 10.3390/microorganisms14061232 PMC13304343 42354857

[31] Vargas-Del Río C, Urrea J, Parra-Calisto A. The SPI-6 T6SS gene cluster from Salmonella Tennessee encodes a new antibacterial nuclease effector protein. Front Microbiol. 2026;17:1794835. doi: 10.3389/fmicb.2026.1794835 PMC13224477 42232912

[32] Liu J, Liu J, Feng C. Divergent roles of hcp genes in Salmonella typhimurium T6SS shape gut microbiota dysbiosis during infection. Microbiol Spectr. 2026;14(4):e0222525. doi: 10.1128/spectrum.02225-25 PMC13055400 41757948

[33] Sui C, Qiao H. The type VI secretion system in enteric pathogen colonization: molecular mechanisms, ecological dynamics, and therapeutic potential. Front Microbiol. 2026;17:1809019. doi: 10.3389/fmicb.2026.1809019 PMC13008689 41883797

[34] Zhao S, Xu Q, Cui Y. Salmonella effector SopB reorganizes cytoskeletal vimentin to maintain replication vacuoles for efficient infection. Nat Commun. 2023;14(1):478. doi: 10.1038/s41467-023-36123-w PMC9885066 36717589

[35] Pilar AVC, Reid-Yu SA, Cooper CA. GogB is an anti-inflammatory effector that limits tissue damage during Salmonella infection through interaction with human FBXO22 and Skp1. PLOS Pathog. 2012;8(6):e1002773. doi: 10.1371/journal.ppat.1002773 PMC3386239 22761574

[36] Coombes BK, Wickham ME, Brown NF. Genetic and molecular analysis of GogB, a phage-encoded type III-secreted substrate in *Salmonella enterica* serovar typhimurium with autonomous expression from its associated phage. J Mol Biol. 2005;348(4):817–830. doi: 10.1016/j.jmb.2005.03.024 15843015

[37] Ammendola S, Secli V, Pacello F. Salmonella Typhimurium and *Pseudomonas aeruginosa* respond differently to the Fe chelator deferiprone and to some novel deferiprone derivatives. IJMS. 2021;22(19):10217. doi: 10.3390/ijms221910217 PMC8508819 34638558

[38] Bharathan G, Mundra S, Darwich DM. Regulation of iron metabolism is critical for the survival of Salmonella Typhimurium in pasteurized milk. Food Microbiol. 2023;115:104326. doi: 10.1016/j.fm.2023.104326 37567619

[39] Ilari A, Pescatori L, Di Santo R. *Salmonella enterica* serovar Typhimurium growth is inhibited by the concomitant binding of Zn(II) and a pyrrolyl-hydroxamate to ZnuA, the soluble component of the ZnuABC transporter. Biochim Biophys Acta (BBA) - Gener Subj. 2016;1860(3):534–541. doi: 10.1016/j.bbagen.2015.12.006 26691136

[40] Cui X, Wang Y, Liu Z. Ginsenoside Ro improves Salmonella Typhimurium-induced colitis through inhibition of the virulence factors SopB and SopE2 via the RAC1/CDC42/ARP2/3 pathway. Faseb J. 2024;38(24):e70282. doi: 10.1096/fj.202401712R PMC11695707 39734277

[41] Wu A, Liang C, Chen W. ZnO-Cu/Mn nanozyme for rescuing the intestinal homeostasis in Salmonella-induced colitis. J Nanobiotechnol. 2025;23(1):225. doi: 10.1186/s12951-025-03283-4 PMC11924796 40114178

[42] Jennewein J, Matuszak J, Walter S. Low-oxygen tensions found in Salmonella-infected gut tissue boost Salmonella replication in macrophages by impairing antimicrobial activity and augmenting Salmonella virulence. Cell Microbiol. 2015;17(12):1833–1847. doi: 10.1111/cmi.12476 26104016

[43] Kelly CJ, Colgan SP. Breathless in the gut: implications of luminal O2 for microbial pathogenicity. Cell Host Microbe. 2016;19(4):427–428. doi: 10.1016/j.chom.2016.03.014 PMC5256639 27078062

[44] Wagner C, Hensel M. Adhesive mechanisms of *Salmonella enterica*. Adv Exp Med Biol. 2011;715:17–34.doi: 10.1007/978-94-007-0940-9_2 21557055

[45] Lopez CA, Rivera-Chávez F, Byndloss MX. The periplasmic nitrate reductase NapABC supports luminal growth of *Salmonella enterica* serovar Typhimurium during colitis. Infect Immun. 2015;83(9):3470–3478. doi: 10.1128/IAI.00351-15 PMC4534643 26099579

[46] Thiennimitr P, Winter SE, Winter MG. Intestinal inflammation allows Salmonella to use ethanolamine to compete with the microbiota. Proc Natl Acad Sci USA. 2011;108(42):17480–17485. doi: 10.1073/pnas.1107857108 PMC3198331 21969563

[47] Paul JK, Azmal M, Haque ASNB. Unlocking the secrets of the human gut microbiota: comprehensive review on its role in different diseases. World J Gastroenterol. 2025;31(5):99913. doi: 10.3748/wjg.v31.i5.99913 PMC11718612 39926224

[48] Wang X, He L, Dong Y. Mucosa-associated bacteria and metabolites in inflammatory bowel disease: from inside to insight. NPJ Biofilms Microbiomes. 2026;12(1):35. doi: 10.1038/s41522-025-00887-4 PMC12877147 41617703

[49] Baird-Parker AC. Foodborne salmonellosis. Lancet. 1990;336(8725):1231–1235. doi: 10.1016/0140-6736(90)92844-8 1978081

[50] Bescucci DM, Moote PE, Ortega Polo R. *Salmonella enterica* serovar Typhimurium temporally modulates the enteric microbiota and host responses to overcome colonization resistance in swine. Appl Environ Microbiol. 2020;86(21). doi: 10.1128/AEM.01569-20 PMC7580545 32859592

[51] Ahmad S, Ali S, Ur Rehman A. Scfas’ pleiotropic role in pathogenesis and salutogenesis: mechanisms in exacerbation and regulation of inflammation and fibrosis from gut to host. J Med Microbiol. 2026;75(1). doi: 10.1099/jmm.0.002117 PMC12856160 41609721

[52] Mukherjee D, Noor S, Mukherjee T. Intracellular formate modulates a motility-invasion switch in Salmonella Typhimurium. PLOS Pathog. 2025;21(9):e1013453. doi: 10.1371/journal.ppat.1013453 PMC12410710 40906700

[53] Lamas A, Regal P, Vázquez B. Short chain fatty acids commonly produced by gut microbiota influence *Salmonella enterica* motility, biofilm formation, and gene expression. Antibiotics (Basel). 2019;8(4):265. doi: 10.3390/antibiotics8040265 PMC6963744 31847278

[54] Hung CC, Garner CD, Slauch JM. The intestinal fatty acid propionate inhibits Salmonella invasion through the post-translational control of HilD. Mol Microbiol. 2013;87(5):1045–1060. doi: 10.1111/mmi.12149 PMC3581741 23289537

[55] Singh S, Koo OK. A comprehensive review exploring the protective role of specific commensal gut bacteria against Salmonella. Pathogens. 2024;13(8):642. doi: 10.3390/pathogens13080642 PMC11356920 39204243

[56] Zhang S, Liu T, Zhou X. Isolation of *Lactiplantibacillus plantarum* for treatment of Salmonella infection in mice. Lett Appl Microbiol. 2024;77(12). doi: 10.1093/lambio/ovae114 39567860

[57] Song J, Li Q, Everaert N. Dietary inulin supplementation modulates short-chain fatty acid levels and cecum microbiota composition and function in chickens infected with Salmonella. Front Microbiol. 2020;11:584380. doi: 10.3389/fmicb.2020.584380 PMC7793945 33424783

[58] Rivera-Chávez F, Zhang L, Faber F. Depletion of butyrate-producing clostridia from the gut microbiota drives an aerobic luminal expansion of Salmonella. Cell Host Microbe. 2016;19(4):443–454. doi: 10.1016/j.chom.2016.03.004 PMC4832419 27078066

[59] Furusawa Y, Obata Y, Fukuda S. Commensal microbe-derived butyrate induces the differentiation of colonic regulatory T cells. Nature. 2013;504(7480):446–450. doi: 10.1038/nature12721 24226770

[60] Lv S, Yang W, Sun C. Gut microbiota dysbiosis and amino acid metabolic dysregulation in children with allergic rhinitis complicated by constipation. Microbiol Spectr. 2026;14(4):e0074825. doi: 10.1128/spectrum.00748-25 PMC13055397 41837426

[61] Radlinski LC, Rogers AWL, Bechtold L. Salmonella virulence factors induce amino acid malabsorption in the ileum to promote ecosystem invasion of the large intestine. Proc Natl Acad Sci USA. 2024;121(47):e2417232121. doi: 10.1073/pnas.2417232121 PMC11588050 39546570

[62] Prasad R, Adu-Rutledge Y, Ziani B. Indole-3-propionic acid links gut dysfunction to diabetic retinopathy: a biomarker and novel therapeutic approach. Gut. 2026;75(5):949–967. doi: 10.1136/gutjnl-2025-336180 PMC12896133 41198173

[63] Hababag EAC, Cauilan A, Quintero D. Tryptophanase expressed by Salmonella halts breast cancer cell growth in vitro and inhibits production of immunosuppressive kynurenine. Microorganisms. 2023;11(5):1355. doi: 10.3390/microorganisms11051355 PMC10220722 37317329

[64] Lin Z, Feng Y, Chen L. Nanozymes modulate probiotic tryptophan metabolism to prevent Salmonella infection in mammalian models. Nat Microbiol. 2025;10(12):3272–3289. doi: 10.1038/s41564-025-02176-4 41286117

[65] Riazati N, Kable ME, Newman JW. Associations of microbial and indoleamine-2,3-dioxygenase-derived tryptophan metabolites with immune activation in healthy adults. Front Immunol. 2022;13:917966. doi: 10.3389/fimmu.2022.917966 PMC9558171 36248784

[66] Kohli N, Crisp Z, Riordan R. The microbiota metabolite indole inhibits Salmonella virulence: involvement of the PhoPQ two-component system. PLOS ONE. 2018;13(1):e0190613. doi: 10.1371/journal.pone.0190613 PMC5771565 29342189

[67] Li C, Zhang B, Zhang Y. Bacterial anti-toxin HigA dysregulates host intestinal serotonin homeostasis in a T6SS-dependent manner. Cell Rep. 2025;44(9):116212. doi: 10.1016/j.celrep.2025.116212 40892545

[68] Close R, Kremer S, Mitchem M. A sulfotransferase from a gut microbe acts on diverse phenolic sulfate compounds, including acetaminophen sulfate. PNAS Nexus. 2026;5(1):pgaf403. doi: 10.1093/pnasnexus/pgaf403 PMC12809581 41552834

[69] Renaldi R, Wiguna T, Persico A. P-cresol and p-cresyl sulphate boost oxidative stress: a systematic review of recent evidence. Basic Clin Pharma Tox. 2025;137(1):e70065. doi: 10.1111/bcpt.70065 PMC12177445 40534235

[70] Calderón-Guzmán D, Hernández-Islas JL, Espítia Vázquez IR. Effect of toluene and cresols on Na+,K+-ATPase, and serotonin in rat brain. Regul Toxicol Pharmacol. 2005;41(1):1–5. doi: 10.1016/j.yrtph.2004.09.005 15649823

[71] Victoria-Montesinos D, García-Muñoz AM, Mercader-Ros MT. The role of microbial-derived p-cresol in autism spectrum disorder: a systematic review of the gut–brain axis. Clin Nutr ESPEN. 2025;69:535–544. doi: 10.1016/j.clnesp.2025.08.010 40783009

[72] Wolfson RL, Chantranupong L, Saxton RA. Sestrin2 is a leucine sensor for the mTORC1 pathway. Science. 2016;351(6268):43–48. doi: 10.1126/science.aab2674 PMC4698017 26449471

[73] Foerster EG, Mukherjee T, Cabral-Fernandes L. How autophagy controls the intestinal epithelial barrier. Autophagy. 2022;18(1):86–103. doi: 10.1080/15548627.2021.1909406 PMC8865220 33906557

[74] He F, Wu C, Li P. Functions and signaling pathways of amino acids in intestinal inflammation. Biomed Res Int. 2018;2018:1–13. doi: 10.1155/2018/9171905 PMC5846438 29682569

[75] Hsu-Ming W, Naito K, Kinoshita Y. Changes in transcription during recovery from heat injury in Salmonella typhimurium and effects of BCAA on recovery. Amino Acids. 2012;42(6):2059–2066. doi: 10.1007/s00726-011-0934-y 21584761

[76] Zhenyukh O, Civantos E, Ruiz-Ortega M. High concentration of branched-chain amino acids promotes oxidative stress, inflammation and migration of human peripheral blood mononuclear cells via mTORC1 activation. Free Radical Biol Med. 2017;104:165–177. doi: 10.1016/j.freeradbiomed.2017.01.009 28089725

[77] Yang X, Stein KR, Hang HC. Anti-infective bile acids bind and inactivate a Salmonella virulence regulator. Nat Chem Biol. 2023;19(1):91–100. doi: 10.1038/s41589-022-01122-3 PMC9805502 36175659

[78] Chiang JYL. Bile acid metabolism and signaling. Compr Physiol. 2013;3(3):1191–1212. doi: 10.1002/j.2040-4603.2013.tb00517.x PMC4422175 23897684

[79] Inagaki T, Choi M, Moschetta A. Fibroblast growth factor 15 functions as an enterohepatic signal to regulate bile acid homeostasis. Cell Metab. 2005;2(4):217–225. doi: 10.1016/j.cmet.2005.09.001 16213224

[80] Agus A, Clément K, Sokol H. Gut microbiota-derived metabolites as central regulators in metabolic disorders. Gut. 2021;70(6):1174–1182. doi: 10.1136/gutjnl-2020-323071 PMC8108286 33272977

[81] Inagaki T, Moschetta A, Lee Y-K. Regulation of antibacterial defense in the small intestine by the nuclear bile acid receptor. Proc Natl Acad Sci USA. 2006;103(10):3920–3925. doi: 10.1073/pnas.0509592103 PMC1450165 16473946

[82] Ridlon JM, Kang DJ, Hylemon PB. Bile salt biotransformations by human intestinal bacteria. J Lipid Res. 2006;47(2):241–259. doi: 10.1194/jlr.R500013-JLR200 16299351

[83] Sato H, Macchiarulo A, Thomas C. Novel potent and selective bile acid derivatives as TGR5 agonists: biological screening, Structure−Activity relationships, and molecular modeling studies. J Med Chem. 2008;51(6):1831–1841. doi: 10.1021/jm7015864 18307294

[84] Bernstein H, Bernstein C, Payne CM. Bile acids as carcinogens in human gastrointestinal cancers. Mutat Res/Rev Mutat Res. 2005;589(1):47–65. doi: 10.1016/j.mrrev.2004.08.001 15652226

[85] Prouty AM, Gunn JS. Comparative analysis of *Salmonella enterica* serovar Typhimurium biofilm formation on gallstones and on glass. Infect Immun. 2003;71(12):7154–7158. doi: 10.1128/IAI.71.12.7154-7158.2003 PMC308894 14638807

[86] Hughes DT, Sperandio V. Inter-kingdom signalling: communication between bacteria and their hosts. Nat Rev Microbiol. 2008;6(2):111–120. doi: 10.1038/nrmicro1836 PMC2667375 18197168

[87] Fiorucci S, Distrutti E. Bile acid-activated receptors, intestinal microbiota, and the treatment of metabolic disorders. Trends Mol Med. 2015;21(11):702–714. doi: 10.1016/j.molmed.2015.09.001 26481828

[88] Foppa C, Rizkala T, Repici A. Microbiota and IBD: current knowledge and future perspectives. Digestive Liver Dis. 2024;56(6):911–922. doi: 10.1016/j.dld.2023.11.015 38008696

[89] Kotla NG, Rochev Y. IBD disease-modifying therapies: insights from emerging therapeutics. Trends Mol Med. 2023;29(3):241–253. doi: 10.1016/j.molmed.2023.01.001 36720660

[90] Shan Y, Lee M, Chang EB. The gut microbiome and inflammatory bowel diseases. Annu Rev Med. 2022;73(1):455–468. doi: 10.1146/annurev-med-042320-021020 PMC10012812 34555295

[91] Quaglio AEV, Grillo TG, Oliveira ECSD. Gut microbiota, inflammatory bowel disease and colorectal cancer. World J Gastroenterol. 2022;28(30):4053–4060. doi: 10.3748/wjg.v28.i30.4053 PMC9403435 36157114

[92] Mentella MC, Scaldaferri F, Pizzoferrato M. Nutrition, IBD and gut microbiota: a review. Nutrients. 2020;12(4):944. doi: 10.3390/nu12040944 PMC7230231 32235316

[93] Lee M, Chang EB. Inflammatory bowel diseases (IBD) and the microbiome—searching the crime scene for clues. Gastroenterology. 2021;160(2):524–537. doi: 10.1053/j.gastro.2020.09.056 PMC8098834 33253681

[94] Sugihara K, Kamada N. Diet–microbiota interactions in inflammatory bowel disease. Nutrients. 2021;13(5):1533. doi: 10.3390/nu13051533 PMC8147260 34062869

[95] Hayase E, Hayase T, Jamal MA. Mucus-degrading Bacteroides link carbapenems to aggravated graft-versus-host disease. Cell. 2022;185(20):3705–3719.e14. doi: 10.1016/j.cell.2022.09.007 PMC9542352 36179667

[96] Schwerdtfeger LA, Montini F, Chitnis T. Faecal mucoprotein MUC2 is decreased in multiple sclerosis and is associated with mucin degrading bacteria. EBioMedicine. 2025;116:105721. doi: 10.1016/j.ebiom.2025.105721 PMC12134549 40344717

[97] Johansson MEV, Gustafsson JK, Holmén-Larsson J. Bacteria penetrate the normally impenetrable inner colon mucus layer in both murine colitis models and patients with ulcerative colitis. Gut. 2014;63(2):281–291. doi: 10.1136/gutjnl-2012-303207 PMC3740207 23426893

[98] Liang L, Liu L, Zhou W. Gut microbiota-derived butyrate regulates gut mucus barrier repair by activating the macrophage/WNT/ERK signaling pathway. Clin Sci. 2022;136(4):291–307. doi: 10.1042/CS20210778 35194640

[99] Suzuki T. Regulation of the intestinal barrier by nutrients: the role of tight junctions. Anim Sci J. 2020;91(1):e13357. doi: 10.1111/asj.13357 PMC7187240 32219956

[100] Christovich A, Luo XM. Gut microbiota, Leaky gut, and autoimmune diseases. Front Immunol. 2022;13:946248. doi: 10.3389/fimmu.2022.946248 PMC9271567 35833129

[101] Peng L, Li Z-R, Green RS. Butyrate enhances the intestinal barrier by facilitating tight junction assembly via activation of AMP-activated protein kinase in caco-2 cell monolayers. J Nutr. 2009;139(9):1619–1625. doi: 10.3945/jn.109.104638 PMC2728689 19625695

[102] Schneemann M, Heils L, Moos V. A colonic organoid model challenged with the large toxins of *Clostridioides difficile* TcdA and TcdB exhibit deregulated tight junction proteins. Toxins (Basel). 2023;15(11):643. doi: 10.3390/toxins15110643 PMC10674794 37999506

[103] Boyle EC, Brown NF, Finlay BB. *Salmonella enterica* serovar Typhimurium effectors SopB, SopE, SopE2 and SipA disrupt tight junction structure and function. Cell Microbiol. 2006;8(12):1946–1957. doi: 10.1111/j.1462-5822.2006.00762.x 16869830

[104] Sharma B, Agriantonis G, Twelker K. Gut microbiota serves as a crucial Independent Biomarker in inflammatory bowel disease (IBD). Int J Mol Sci. 2025;26(6):2503. doi: 10.3390/ijms26062503 PMC11942158 40141145

[105] Tie Y, Huang Y, Chen R. Current insights on the roles of gut microbiota in inflammatory bowel disease-associated extra-intestinal manifestations: pathophysiology and therapeutic targets. Gut Microbes. 2023;15(2):2265028. doi: 10.1080/19490976.2023.2265028 PMC10572083 37822139

[106] Masaadeh AH, Eletrebi M, Parajuli B. Human colitis-associated colorectal carcinoma progression is accompanied by dysbiosis with enriched pathobionts. Gut Microbes. 2025;17(1):2479774. doi: 10.1080/19490976.2025.2479774 PMC11917176 40094201

[107] Sahoo DK, Heilmann RM, Paital B. Oxidative stress, hormones, and effects of natural antioxidants on intestinal inflammation in inflammatory bowel disease. Front Endocrinol. 2023;14:1217165. doi: 10.3389/fendo.2023.1217165 PMC10493311 37701897

[108] Ma X, Chen J, Wang F. High fructose consumption aggravates inflammation by promoting effector T cell generation via inducing metabolic reprogramming. Sig Transduct Target Ther. 2025;10(1):271. doi: 10.1038/s41392-025-02359-9 PMC12379281 40854873

[109] Yu Y, Bu Y, Shen C. Bacteriocin-transport-inspired oral peptide-probiotic delivery ameliorates IBD complications via autophagy and gut homeostasis. Sci Adv. 2026;12(1):eadz9069. doi: 10.1126/sciadv.adz9069 PMC12758525 41481708

[110] Guo XY, Liu XJ, Hao JY. Gut microbiota in ulcerative colitis: insights on pathogenesis and treatment. J Dig Dis. 2020;21(3):147–159. doi: 10.1111/1751-2980.12849 32040250

[111] Zhang J, Mak JWY, Ng SC. Gut microbiome in IBD: past, present and the future. Gut. 2026;75(2):398–410. doi: 10.1136/gutjnl-2025-335626 41219057

[112] Chen S, Liu F, Han X. Exposure to bovine viral diarrhea virus disrupts intestinal barrier function via NLRP3/Caspase-1-mediated pyroptosis and gut microbiota dysbiosis. J Agric Food Chem. 2025;73(36):22384–22396. doi: 10.1021/acs.jafc.5c04854 40884832

[113] Vance RE. The NAIP/NLRC4 inflammasomes. Curr Opin Immunol. 2015;32:84–89. doi: 10.1016/j.coi.2015.01.010 PMC4336817 25621709

[114] Thurston TL, Matthews SA, Jennings E. Growth inhibition of cytosolic Salmonella by caspase-1 and caspase-11 precedes host cell death. Nat Commun. 2016;7(1):13292. doi: 10.1038/ncomms13292 PMC5097160 27808091

[115] Yu T, Luo L, Xue J. Gut microbiota–NLRP3 inflammasome crosstalk in metabolic dysfunction-associated steatotic liver disease. Clin Res Hepatol Gastroenterol. 2024;48(8):102458. doi: 10.1016/j.clinre.2024.102458 39233138

[116] Zeng Y, Liu X, Yi Q. Free total rhubarb anthraquinones protect intestinal mucosal barrier of SAP rats via inhibiting the NLRP3/caspase-1/GSDMD pyroptotic pathway. J Ethnopharmacol. 2024;326:117873. doi: 10.1016/j.jep.2024.117873 38346523

[117] Bierschenk D, Monteleone M, Moghaddas F. The Salmonella pathogenicity island-2 subverts human NLRP3 and NLRC4 inflammasome responses. J Leukoc Biol. 2019;105(2):401–410. doi: 10.1002/JLB.MA0318-112RR 30368901

[118] Clare B. Inflammasome activation by Salmonella. Curr Opin Microbiol. 2021;64:27–32. doi: 10.1016/j.mib.2021.09.004 34563937

[119] Gupta S, Cassel SL, Sutterwala FS. Regulation of the NLRP3 inflammasome by autophagy and mitophagy. Immunol Rev. 2025;329(1):e13410. doi: 10.1111/imr.13410 PMC12477832 39417249

[120] Xue S, Xue Y, Dou D. Kui Jie Tong ameliorates ulcerative colitis by regulating gut microbiota and NLRP3/Caspase-1 classical pyroptosis signaling pathway. Dis Markers. 2022;2022:1–15. doi: 10.1155/2022/2782112 PMC9273439 35832643

[121] Jia J, Liu Y, Wang D. Ulcerative colitis: signaling pathways, therapeutic targets and interventional strategies. Sig Transduct Target Ther. 2026;11(1):51. doi: 10.1038/s41392-025-02345-1 PMC12890998 41667440

[122] Schroder K, Zhou R, Tschopp J. The NLRP3 inflammasome: a sensor for metabolic danger? Science. 2010;327(5963):296–300. doi: 10.1126/science.1184003 20075245

[123] Liu D, Zhou C, Wang X. Nucleotide metabolic rewiring enables NLRP3 inflammasome hyperactivation in obesity. Science. 2026;391(6782):eadq9006. doi: 10.1126/science.adq9006 PMC13034487 41538457

[124] Kim JJ, Jo EK. NLRP3 inflammasome and host protection against bacterial infection. J Korean Med Sci. 2013;28(10):1415–1423. doi: 10.3346/jkms.2013.28.10.1415 PMC3792593 24133343

[125] Kayagaki N, Stowe IB, Lee BL. Caspase-11 cleaves gasdermin D for non-canonical inflammasome signalling. Nature. 2015;526(7575):666–671. doi: 10.1038/nature15541 26375259

[126] Crowley SM, Han X, Allaire JM. Intestinal restriction of Salmonella Typhimurium requires caspase-1 and caspase-11 epithelial intrinsic inflammasomes. PLOS Pathog. 2020;16(4):e1008498. doi: 10.1371/journal.ppat.1008498 PMC7179941 32282854

[127] Ren J, Sun W, Wang S. Activation of IL-1 and IL-18 induced by mycobacterium neoaurum and bacillus calmette-guérin are dependent on the NLRP3 and AIM2 inflammasome. Vet Res Commun. 2026;50(2):89. doi: 10.1007/s11259-025-11023-0 41505008

[128] He WT, Wan H, Hu L. Gasdermin D is an executor of pyroptosis and required for interleukin-1β secretion. Cell Res. 2015;25(12):1285–1298. doi: 10.1038/cr.2015.139 PMC4670995 26611636

[129] Kayagaki N, Wong MT, Stowe IB. Noncanonical inflammasome activation by intracellular LPS independent of TLR4. Science. 2013;341(6151):1246–1249. doi: 10.1126/science.1240248 23887873

[130] Han X, Allaire JM, Crowley SM. Inflammasome activation links enteric Salmonella Typhimurium infection to a rapid, cytokine-dependent increase in intestinal mucin release. Gut Microbes. 2024;16(1):2413372. doi: 10.1080/19490976.2024.2413372 PMC11497969 39428744

[131] Bumann D, Schothorst J. Intracellular Salmonella metabolism. Cell Microbiol. 2017;19(10):e12766. doi: 10.1111/cmi.12766 28672057

[132] Jarret A, Jackson R, Duizer C. Enteric nervous system-derived IL-18 orchestrates mucosal barrier immunity. Cell. 2020;180(1):50–63.e12. doi: 10.1016/j.cell.2019.12.016 PMC7339937 31923399

[133] Liu Y, Gao J, Chen L. Lithocholic acid ameliorates ulcerative colitis via the PXR/TLR4/NF-κB/NLRP3 signaling pathway and gut microbiota modulation. Cell Mol Life Sci. 2025;82(1):336. doi: 10.1007/s00018-025-05834-2 PMC12411392 40905977

[134] Holtmann TM, Inzaugarat ME, Knorr J. Bile acids activate NLRP3 inflammasome, promoting murine liver inflammation or fibrosis in a cell type-specific manner. Cells. 2021;10(10):2618. doi: 10.3390/cells10102618 PMC8534222 34685598

[135] Wu K, Yuan Y, Yu H. The gut microbial metabolite trimethylamine N-oxide aggravates GVHD by inducing M1 macrophage polarization in mice. Blood. 2020;136(4):501–515. doi: 10.1182/blood.2019003990 PMC7378459 32291445

[136] Zhang ZY, Guo X-L, Liu JTY. Conjugated bile acids alleviate acute pancreatitis through inhibition of TGR5 and NLRP3 mediated inflammation. J Transl Med. 2024;22(1):1124. doi: 10.1186/s12967-024-05922-0 PMC11662532 39707318

[137] Chen S, Li Y, Chu B. *Lactobacillus johnsonii* L531 alleviates the damage caused by Salmonella Typhimurium via inhibiting TLR4, NF-κB, and NLRP3 inflammasome signaling pathways. Microorganisms. 2021;9(9):1983. doi: 10.3390/microorganisms9091983 PMC8468631 34576878

[138] Duda-Madej A, Viscardi S, Łabaz JP. Berberine in bowel health: anti-inflammatory and gut microbiota modulatory effects. IJMS. 2025;26(24):12021. doi: 10.3390/ijms262412021 PMC12732474 41465447

[139] Pelyuntha W, Ngasaman R, Yingkajorn M. Phage cocktail administration to reduce Salmonella load in broilers. Res Vet Sci. 2024;169:105163. doi: 10.1016/j.rvsc.2024.105163 38295630

